# Editorial: *In vivo* magnetic resonance imaging of metabolic disorders

**DOI:** 10.3389/fendo.2025.1713999

**Published:** 2025-11-13

**Authors:** An Tang, Martin Lepage, Guy A. Rutter, André C. Carpentier

**Affiliations:** 1 Department of Radiology, Radiation Oncology and Nuclear Medicine, Université de Montréal, Montréal, QC, Canada; 2 Department of Radiology, Centre hospitalier de l’Université de Montréal (CHUM), Montréal, QC, Canada; 3 Centre de recherche du Centre hospitalier de l’Université de Montréal (CRCHUM), Montréal, QC, Canada; 4 Department of Medical Imaging and Radiation Sciences, Université de Sherbrooke, Sherbrooke, QC, Canada; 5 Centre de recherche du Centre hospitalier universitaire de Sherbrooke (CRCHUS), Sherbrooke, QC, Canada; 6 Section of Cell Biology and Functional Genomics, Division of Diabetes, Endocrinology and Metabolism, Faculty of Medicine, Department of Metabolism, Digestion and Reproduction, Faculty of Medicine, Imperial College London, Hammersmith Hospital, London, United Kingdom; 7 Research Institute of the McGill University Health Centre, Montreal, QC, Canada; 8 Lee Kong Chian School of Medicine, Nanyang Technological College, Singapore, Singapore; 9 Department of Medicine, Université de Sherbrooke, Sherbrooke, QC, Canada

**Keywords:** magnetic resonance imaging (MRI), magnetic resonance spectroscopy (MRS), metabolic disorders, adipose tissue, diabetes, metabolism

## 1 Introduction

Metabolic health depends on the balance between energy expenditure through oxidation, substrate supply, and substrate storage. When this balance is disrupted, for example when substrate availability chronically exceeds energy expenditure, fatty acids and carbohydrates may be stored as triglycerides in adipose tissue. The subcutaneous and visceral adipose tissues serve as the body’s long-term energy reserve (1). Excess triglycerides may alternately be stored in the liver (2), skeletal muscle (3), and in the pancreas (4), leading to ectopic lipid deposition and lipotoxicity which interfere with insulin signaling (5). In the liver, steatosis leads to the development of metabolic dysfunction-associated steatotic liver disease (MASLD) and metabolic dysfunction-associated steatohepatitis (MASH) (6). In the endocrine pancreas, lipotoxicity plays a key role in reduced beta-cell function and mass and impaired beta-cell vascularization (7). Further, enlarged adipocytes secrete pro-inflammatory cytokines which further worsen insulin resistance and type 2 diabetes (8). A major challenge in research of metabolic disorders is the ability to evaluate several organ systems and phenomena (*e.g.*, fat metabolism, glycogen metabolism, blood flow, oxygenation) concurrently to better understand the dynamic evolution of these diseases *in vivo*.

Magnetic resonance imaging (MRI) and magnetic resonance spectroscopy (MRS) are nonionizing techniques that display outstanding anatomical detail with high spatial resolution and numerous tissue contrast mechanisms. Magnetic resonance techniques for fat quantification rely on difference in resonance frequencies between water and fat proton signals to quantitatively measure the proton density fat fraction (9, 10). Other relaxometry techniques measure relaxation constants such as T1 and T2 to differentiate normal and pathologic tissues. Advanced MRI techniques also allow measurement of tissue blood flow, either with gadolinium-based contrast agents (*e.g.*, dynamic contrast-enhanced imaging) or without contrast agents (*e.g.*, using arterial spin labelling) (11–13). Further, MRS can resolve distinct fat peaks based on their resonance frequencies and provide information on the chemical structure of triglycerides to determine fatty acid composition by differentiating saturated, monounsaturated, and polyunsaturated fatty acids fractions (14). Because of the abundance of hydrogen in the body leading to high signal, most clinical magnetic resonance techniques focus on hydrogen (^1^H) imaging or spectroscopy. With the use of specialized hardware and sequences, MRS of other nuclei to provide insights on metabolic pathways using isotopes such as the hydrogen isotope deuterium (^2^H), carbon isotope (^13^C), or phosphorus (^31^P). Remarkably, these techniques can achieve noninvasive, quantitative, repeated, and longitudinal assessment of several metabolic pathways concomitantly within the same examination by using different sequences.

## 2 Key contributions to this Research Topic

In this Research Topic, Kupriyanova and Schrauwen-Hinderling describe current practice and recent advances in metabolic research in their review article entitled ‘Advances in *in vivo* magnetic resonance spectroscopy for metabolic disorders’. The authors describe potential applications of MRS, specifically in the field of obesity, insulin resistance and diabetes. For example, ^31^P-MRS can provide *in vivo*, organ-specific, measurements of oxidative or non-oxidative metabolism; ^1^H-MRS of lipid content and type of fatty acids; and ^13^C-MRS of glycogen concentration and turnover. Their review also highlights advantages of MRS such has real-time dynamic information to investigate metabolism during physiological challenges, non-invasive nature which allows longitudinal monitoring of treatment response, and alleviate the need for biopsies. The review briefly mentions strategies to mitigate motion which can affect the quality of MRS and strategies for motion correction.

Garcia et al. describe compressed sensing techniques, an acceleration method for MRI signal acquisition translated to MRS, to evaluate energy metabolism *in vivo* using ^31^P-MRS and MRS imaging in their original research article entitled ‘Assessment of reconstruction accuracy for under-sampled ^31^P-MRS data using compressed sensing and a low rank Hankel matrix completion approach’. By using this approach, the team was able to shorten the long acquisition times typically required to measure metabolites such as phosphocreatine and inorganic phosphate in brain and skeletal muscle tissue. They analyzed factors that influence the quality of the signal reconstruction. Their findings revealed that reconstruction accuracy is influenced by the selection of samples and their density rather than the undersampling factor. Future work will require quantitative assessment to validate the fidelity of the proposed method for reconstructing individual spectral components.

Mori et al. investigated the potential impact of a sodium-glucose cotransporter 2 (SGLT2) inhibitor on kidney oxygenation in their original research article entitled ‘Effects of canagliflozin on kidney oxygenation evaluated using blood oxygenation level-dependent MRI in patients with type 2 diabetes’. With repeated mapping of T2*, which is related to blood oxygenation, (using blood oxygenation level-dependent, BOLD MRI) the authors found that short-term canagliflozin treatment was associated with higher T2* values indicating good levels of tissue oxygenation. However, the results of this single-arm study will need to be validated in future studies with a control group. Increase in oxygenation induced by the administration of SGLT2 inhibitor in type 2 diabetes may improve kidney outcomes, as kidney injury has been thought to be induced by hypoxic damage (15).

Xie et al. investigated two MRI techniques in their original research article entitled ‘T1 mapping combined with arterial spin labeling MRI to identify renal injury in patients with liver cirrhosis’. The authors found lower T1 values in the renal cortex and medulla of normal controls than in cirrhotic participants. They also proposed a classification and regression tree model incorporating cortical T1 values and cortical renal blood flow derived from arterial spin labelling to identify renal injury. Their findings suggest that renal T1 mapping may be used for early detection of renal injury in the setting of cirrhosis. This proof-of-concept study will also require validation against other techniques such as para-aminohippurate clearance for assessing effective renal blood flow, or histopathology for confirming the presence of renal injury prior to clinical adoption.

Finally, Li et al. evaluated alterations in marrow fat content in their original research article entitled ‘Associations of marrow fat fraction with MRI based trabecular bone microarchitecture in first-time diagnosed type 1 diabetes mellitus’. The authors performed a case-control study in adults diagnosed with type 1 diabetes mellitus and age-and sex-matched healthy volunteers. They evaluated the trabecular microarchitecture of the tibia with X-ray absorptiometry and fat fraction by MRI. While bone density was similar between the two groups, the trabecular separation, volume, number, and fat fraction were higher in type 1 diabetes mellitus than in controls. Their findings highlight alterations in trabecular bone microarchitecture and expansion of marrow adiposity in type 1 diabetes mellitus. These measurements may be further investigated as quantitative tools for assessing diabetic bone fragility.

A common thread among these contributions is the use of various magnetic resonance techniques to assess organ-specific manifestations of disease without the need for invasive tissue sampling. Collectively, the articles in this Research Topic provide insights into metabolic disorders using noninvasive MRS and MRI techniques. By focusing on tissue properties *in vivo*, these articles highlighted the potential of these quantitative techniques for assessing manifestations of disease in diabetes, kidney injury, or bone fragility. The diversity of tissue contrast mechanisms exploited to differentiate normal and pathologic tissue and the variety of organs assessable with magnetic resonance techniques showcase the versatility of this modality. Importantly, magnetic resonance techniques can assess fatty tissue content, type (saturated vs. unsaturated fatty acids), and distribution. A major unmet need in this field is the development of an integrated panel of molecular imaging tools capable of assessing dysregulation of energy and fatty acid metabolism across key organs, including adipose tissue (fat and brown), liver, pancreas, brain, and skeletal muscle. Moving forward, we anticipate that magnetic resonance techniques will help identify critical organ-specific pathogenic biomarkers that will lead to better, individually tailored strategies for clinical management.
